# Blood vessel imaging using radiofrequency-induced second harmonic acoustic response

**DOI:** 10.1038/s41598-018-33732-0

**Published:** 2018-10-19

**Authors:** Yuanhui Huang, Stephan Kellnberger, George Sergiadis, Vasilis Ntziachristos

**Affiliations:** 1 Helmholtz Zentrum München, Institute for Biological and Medical Imaging (IBMI), Neuherberg, D-85764 Germany; 2 Technische Universität München, Chair for Biological Imaging, München, D-81675 Germany; 3 Cardiovascular Research Center, Cardiology Division, Massachusetts General Hospital, Harvard Medical School, Boston, Massachusetts 02114 USA; 4 Aristotle University of Thessaloniki, School of Electrical and Computer Engineering, Thessaloniki, 54124 Greece; 5 Chinese Academy of Sciences, Suzhou Institute of Biomedical Engineering and Technology, Suzhou, 215163 China

## Abstract

We introduce a contrast mechanism for visualizing blood vessels based on radiofrequency-induced second harmonic acoustic (RISHA) signals sensing blood conductivity. We develop a novel imaging system using commonly available inexpensive components, and demonstrate *in vivo* RISHA visualization of blood vessels based on low-power quasi-continuous radiofrequency excitation of tissue at frequencies of a few MHz. We show how the novel approach also implicitly enables radiofrequency-induced passive ultrasound imaging and can be readily applied to non-invasive imaging of blood vessels *ex vivo* and *in vivo*. We discuss the implications of non-invasive conductivity measurements in the context of biomedical applications.

## Introduction

Vascular imaging plays a central role in the assessment of blood vessels and can be used to diagnose diseases related to abnormal blood flow^1–3^. Different contrast mechanisms have been explored for non-invasive portable imaging of tissue vasculature. While existing methods exploit different properties of blood to visualize vasculature, the underlying contrast mechanisms inherently limit the application of these methods in vascular imaging. Ultrasonography exploits differences in the reflection of ultrasound (US) waves between vascular structures and surrounding tissue, allowing the visualization of large blood vessels as hypoechoic regions^4,5^. Such a method can also identify vessel functionality by detecting Doppler shifts in emitted ultrasonic frequencies because of blood flow^5–7^. Optoacoustic imaging^7,8^, based on the strong optical absorption by hemoglobin, also allows visualization of vessels, typically over a larger diameter span than conventional ultrasonography^9,10^. Optical coherence tomography^11^ has also been considered for imaging fine vasculature, based on variations of reflected light due to blood flow, but it is suited for visualizing only superficial vessels at depths of about 400 microns. In contrast to using mechanical waves or optical energy, higher-energy photons do not provide adequate contrast for blood vessels. Consequently, X-ray computed tomography^12,13^ requires the use of iodine-based contrast agents. Similarly, magnetic resonance angiography^14^ requires gadolinium-based contrast agents for vasculature imaging.

In this work, we explore a new, agent-free contrast mechanism for non-invasive blood vessel imaging. We show for the first time that radiofrequency (RF) energy can be used for high-resolution label-free blood vessel visualization based on difference in electrical conductivity between blood and surrounding tissues. We explain the basis of radiofrequency-induced second harmonic acoustic (RISHA) generation and explain how quasi-continuous RF waves in the MHz range, delivered by energy coupling in the near-field, can be employed to visualize vasculature *in vivo* and *ex vivo*. We develop a RISHA sensing modality and investigate whether RF fields with wavelengths exceeding 10 meters can enable RISHA imaging of small vasculature in mice. We further examine the dependence of blood RISHA response on oxygenation state and demonstrate the implicit ability of the new modality to produce RF-induced passive ultrasound images.

## Results

### Concept of the RISHA signal generation

We have recently^15,16^ shown that electrically conductive materials exposed to continuous wave (CW) radiofrequency excitation at frequency *f*_*RF*_ emit ultrasonic (RISHA) waves at double the frequency of the excitation radiofrequency wave, i.e. 2 × *f*_*RF*_. RISHA waves $p(\overrightarrow{r},\omega )$ at angular frequency *ω* and detected at position $\overrightarrow{r}$ result from the absorption of RF energy by the conductive material. RISHA wave can be described using the thermoacoustic wave equation in the frequency domain, assuming an RF electric field (E-field) source^15^:

1 $$({\nabla }^{2}+{(\frac{\omega }{{{v}}_{{\boldsymbol{s}}}})}^{2}){p}\,(\overrightarrow{{r}},\omega )=\frac{-{j}\omega \beta }{{{C}}_{{p}}}\sigma (\overrightarrow{{r}}){|\hat{{E}}(\overrightarrow{{r}},{\omega }_{0})|}^{2}$$

where ∇^2^ denotes the spatial Laplacian operator, *v*_*s*_ is the acoustic speed in medium, *β* is the thermal expansion coefficient, *C*_*p*_ is the specific heat capacity, $\sigma (\overrightarrow{r})$ is the spatial distribution of electrical conductivity of the medium imaged, $\hat{E}(\overrightarrow{r},{\omega }_{0})$ is the spectral E-field amplitude in the medium, and $j=\sqrt{-1}$. Assuming the applied E-field has an angular frequency *ω*_0_ = 2*πf*_*RF*_, i.e. $E(\overrightarrow{r},t)={\overrightarrow{e}}_{r}{E}_{0}\,\cos \,({\omega }_{0}t)={\overrightarrow{e}}_{r}{E}_{0}\,\cos \,(2\pi {f}_{RF}t)$, the RF power distribution in the Fourier domain can be then calculated as:

2 $${|\hat{{E}}(\overrightarrow{{r}},\omega )|}^{2}=\frac{\pi }{2}{{{E}}_{0}}^{2}{\overrightarrow{{e}}}_{{r}}[\delta (\omega -2{\omega }_{0})+\delta (\omega +2{\omega }_{0})+2\delta (\omega )]$$

Substituting Eq. (2) into Eq. (1) and solving for the RISHA wave $p(\overrightarrow{r},\omega )$ yields a second harmonic acoustic pressure wave with a frequency resonating at two times the E-field frequency *ω* = 2*ω*_0_, i.e. *f*_*RISHA*_ = 2 × *f*_*RF*_. For example, it has been shown^15,16^ that conductive materials absorbing RF energy with *f*_*RF*_ = 3.1 MHz generate RISHA waves at *f*_*RISHA*_ = 2 × *f*_*RF*_ = 6.2 MHz, based on the thermoacoustic effect.

Second harmonic acoustic wave generation in thermoacoustics is universal to all excitation frequencies and not limited to the RF spectrum. While we show second harmonic acoustic waves only at RF frequencies, this phenomenon is also occurring at higher frequencies in the GHz and THz band. However, the detection of GHz and THz acoustic waves is technically challenging due to the unavailability of acoustic detectors in this frequency band and the strong acoustic attenuation. Therefore, as a proof-of-concept study, we chose a relatively low frequency in the RF range for RISHA sensing, because (1) the second harmonic acoustic wave is within the detection bandwidth of our transducers, (2) the acoustic attenuation is moderate as acoustic attenuation increases as an exponential function of frequency, and (3) low frequency RF enables higher penetration depths in tissue.

### RISHA imaging set-up

**Figure 1:**
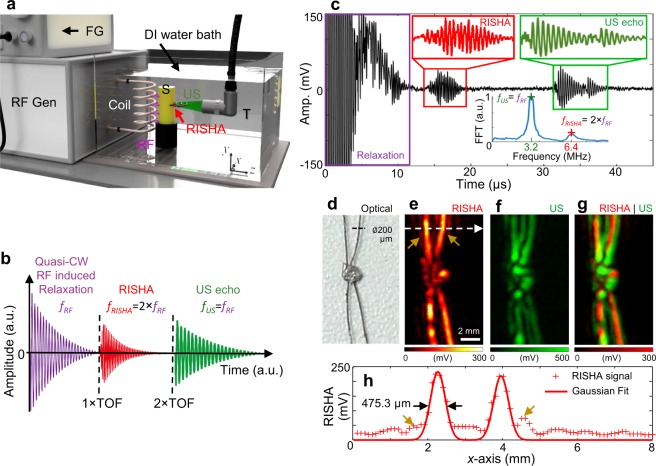
RISHA imaging set-up and tests on blood and copper wires. (**a**) Schematic of RISHA set-up. A function generator (FG) triggers a radiofrequency generator (RF Gen) to produce RF-field bursts. A coil couples RF energy to the samples (S) within its near-field. The induced RISHA waves are detected by an ultrasound transducer (T). The transducer also transmits ultrasound (US) waves due to electromagnetic coupling of the RF field. The coil, sample, and transducer are immersed in de-ionized (DI) water. Translational stages (not shown) scan the transducer in the *xy* plane. (**b**) Temporal signals involved in RISHA sensing. The transducer records at the beginning the relaxation signal during quasi-CW RF excitation induced by electromagnetic coupling (purple, frequency *f*_*RF*_), and the US echoes (green, *f*_*US*_ = *f*_*RF*_) at a time of flight of a roundtrip (2 × TOF). RISHA waves (red, *f*_*RISHA*_ = 2 × *f*_*RF*_) are detected at 1 × TOF. (**c**) RISHA response of mouse blood in a polyethylene tube. The 0–12 µs activity corresponds to the transducer relaxation due to RF coupling. RISHA responses and US echoes have different TOFs. Inset Fourier transform (FFT) shows the differences of their spectral frequency: ultrasound at *f*_*US*_ = *f*_*RF*_ = 3.2 MHz; RISHA at *f*_*RISHA*_ = 2 × *f*_*RF*_ = 6.4 MHz. (**d**–**h**) Dual-mode RISHA/US imaging of copper wires soldered at their crossover point. (**d**) Photograph of the wires. (**e**) RISHA image. The dashed arrow indicates the line scanned in panel h, and brown arrows indicate side lobes due to acoustic interference. (**f**) US image. (**g**) Co-registered RISHA (red) and US (green) images. (**h**) Line profile showing a target-to-background ratio (TBR) of 20 dB and a resolution of 475.3 µm. Scale bar, 2 mm.

We hypothesized that near-field^15–19^, narrowband RF excitation in the low-MHz range can generate RISHA responses from blood and that these responses can be detected and reconstructed into blood vessel images based on differences in electrical conductivity between blood and surrounding tissues. To test this hypothesis, we implemented a RISHA experimental setup consisting of a custom-built coil to couple RF energy to a sample in its near-field (Fig. 1a). In contrast to typical thermoacoustic imaging implementations^20^, which irradiate the sample in the far-field^21,22^ usually at GHz-range frequencies, near-field energy coupling enables optimal RF energy deposition even in the few-MHz range and minimizes the requirement for high-power pulsers^15–19,23^. Figure 1a shows diagrammatically the RISHA imaging set-up (see Methods), which comprises a custom-built 3.2 MHz RF generator emitting 12 µs RF-field bursts at a maximum energy of 765 mJ and repetition rate of 1–50 Hz, a homemade coil to couple energy to samples in its near-field, and a 10-MHz transducer (focus, 25.4 mm; central frequency, 10 MHz; *f*-number, 2) to capture RISHA responses. The coil, samples, and transducer are immersed in de-ionized water (electrical conductivity < 5.5 × 10^−6^ S/m) for optimal RF energy and RISHA wave coupling. The transducer is mounted on a *xy* stage for raster scans with 100-µm step size.

Figure 1b schematically describes the temporal signals involved in RISHA sensing. During an RF excitation, electromagnetic coupling of the RF field to the piezoelectric transducer causes transducer relaxation^15,17,18,24^, which generates pulse/echo US signals approximately 60 dB weaker than the signal generated by a conventional pulser/receiver unit (Model 5077PR, Olympus-Panametrics, USA), as shown in Supplementary Text 1 and Fig. S1. To separate RISHA signal in temporal sequence from the transducer relaxation, we applied the quasi-CW excitation, instead of CW excitation, as the narrowband RF excitation (0.16 MHz bandwidth defined by full-width at half-maximum, FWHM). Likewise, quasi-CW excitation enables also the temporal separation of RISHA and echo US signal that is generated during transducer relaxation. The echo US signal is herein exploited for simultaneous ultrasonography imaging of hyperechoic target based on time-of-flight (TOF) measurements. For instance, by placing the transducer 18 mm away from the sample imaged, RISHA signals can be detected 12 µs after excitation, i.e. 1 × TOF required to traverse the distance from sample to detector, as shown in Fig. 1b,c. As previously demonstrated^15,16^, the RISHA signals oscillate at the second harmonic of the excitation radiofrequency: *f*_*RISHA*_ = 2 × *f*_*RF*_. At 2 × TOF, the detector also records the pulse-echo US signal emitted during its RF-induced relaxation. This US signal exhibits a frequency *f*_*US*_ = *f*_*RF*_. RISHA set-up using quasi-CW excitation enables co-registered dual-mode RISHA/US imaging based on the difference either in frequencies or TOFs measurement (see Methods for details of signal processing and image formation).

### RISHA sensing: whole blood

Figure 1c shows the simultaneous RISHA and RF-induced US signals detected from a polyethylene tube containing mouse blood. The blood sample was excited by the quasi-CW RF-field bursts of fundamental frequency *f*_*RF*_ = 3.2 MHz, and the resulting RISHA waves were detected at the second harmonic of *f*_*RF*_, i.e. *f*_*RISHA*_ = 2 × *f*_*RF*_ = 6.4 MHz, occurring at a TOF of 15–20 µs after the start of the RF emission. At 2 × TOF (30–40 µs) of the same sequence, ultrasound echoes of the tubing walls were detected. The frequency spectra of the generated RISHA waves and the US echoes are shown in the Fourier analysis in the inset of Fig. 1c. This first demonstration of blood sensing based on its electrical conductivity was achieved herein with second harmonic acoustic signals, due to the use of quasi-continuous excitation and near-field coupling.

### RISHA imaging performance

To characterize the RISHA-based blood imaging system and assess its performance, we first analyzed the RISHA responses from a pair of crossed copper wires (200 µm in diameter) soldered together at their crossover point (Fig. 1d). RISHA imaging of the copper wire phantom was performed by scanning the transducer in the *xy* plane in steps of 100 µm. Figure 1e shows the maximum intensity projection (MIP) of RISHA responses of the crossed copper wires, based on RF energy absorption from the wires (see Methods for details of signal processing and image formation). Figure 1f shows the corresponding US echo image, related to the acoustic impedance of the wires. Figure 1g shows the co-registered dual-mode RISHA/US image, illustrating the complementary contrast of the RISHA signal (rendered in red) to the US echo signal (rendered in green). The US image appears as a ‘shadow’ of the RISHA image and does not overlap perfectly, due to the constructive and destructive interference patterns of its narrowband acoustic signals, similar to the effect that was reported by Mohajerani *et al*.^25^ and confirmed by our simulations using k-Wave^12^ (see Methods for k-Wave simulation, Supplementary Text 2 and Fig. S2).

Figure 1h shows a line profile of the RISHA image of copper wires (along white arrow in Fig. 1e), indicating a target-to-background ratio (TBR) of 20 dB in the RISHA image. Deconvolution^26^ of the estimated FWHM by the wire diameter indicates a lateral resolution of 475.3 µm, consistent with the diffraction-limited resolution of 469 µm of the transducer at 6.4 MHz. The achieved lateral resolution is approximately 1/(2.2 × 10^4^) of the 3.2-MHz RF wavelength used, which is 10.5 meters in de-ionized water. This ratio of RISHA resolution to RF wavelength is constant for a given detector *f-*number, which was 2 in our case (see Methods for detailed analysis of imaging resolution).

The axial resolution of RISHA is measured to be ~1.35 mm using the same copper wire due to the relatively long duration of the RF excitation burst, as shown in Supplementary Fig. S3c,d and Text 3. We also note that the axial resolution of RISHA is not limited to 1.35 mm, but can be improved by applying shorter RF-bursts for RISHA signal excitation, and converting RISHA to higher excitation frequencies in the order of 10–100 MHz^17–19^.

To study the penetration depth of our RISHA imaging set-up, we imaged a copper wire of 1 mm in diameter at different depths within chicken muscle (Supplementary Text 4 and Fig. S4). The results suggest that the loss/decay of near-field RF energy in thick conductive tissue has a significant impact on the penetration depth of RISHA imaging. In its present state, the dual-mode RISHA/US imager can visualize conductive materials, such as copper wires, with a RISHA target-to-background ratio of 6 dB to a depth of 25 mm in muscle tissue (ultrasound attenuation in soft tissue^27^ is 0.75 dB/cm/MHz).

### RISHA imaging of blood phantoms

**Figure 2:**
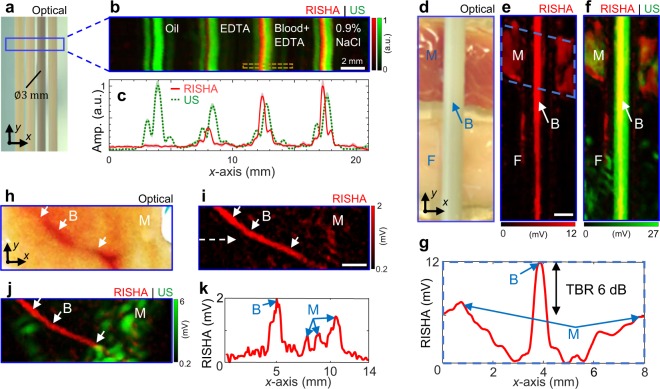
RISHA imaging of blood phantoms. (**a**–**c**) Imaging of polyethylene tubes (*ϕ*3 mm) filled with vegetable oil, an aqueous solution of anticoagulant EDTA, mouse blood containing the same EDTA concentration as the aqueous solution, or 0.9% NaCl. (**a**) Photograph of the tubes. The blue box indicates the scanned region. (**b**) Co-registered RISHA (red) and US (green) images. The dashed brown box is analyzed in Supplementary Fig. S2. The yellowish color is a result of adding red with green showing the overlapping area of the two contrasts. (**c**) Average of signals from panel b along *y*-axis (40 rows/measurements) showing relative amplitudes of RF absorption (solid red) and US echo (dashed green) in *x*-axis. The shaded color patches that is surrounding the average signal show the standard deviation of the measurements. (**d**–**g**) Imaging of a synthetic phantom containing a tube of mouse blood (B) overlaid on side-by-side pieces of porcine fat (F) and muscle (M). (**d**) Photograph of the phantom. (**e**) RISHA image. The dashed blue box indicates the region analyzed in panel g. (**f**) Co-registered RISHA and US images. The yellowish color is a result of adding red with green showing the overlapping area of the two contrasts. (**g**) Average of RISHA signals in the dashed box in panel e, along the *y-*axis. The resulting projection along the *x*-axis shows two-fold higher RF absorption (TBR 6 dB) by blood than by muscle. (**h**–**k**) Imaging of *ex-vivo* chicken tissue containing a blood vessel and muscle. Arrows indicate blood vessel. (**h**) Photograph of the tissue. (**i**) RISHA image. The dashed arrow indicates a line profile analyzed in panel k. (**j**) Co-registered RISHA and US images. (**k**) Line profile of RISHA signals along the dashed arrow in panel i showing two-fold higher RF absorption by blood than by muscle. Scale bars are 2 mm.

Whole blood exhibits good electrical conductivity (0.98 S/m at 3 MHz), suggesting nearly two-fold (6 dB) greater RF absorption than muscle tissue^28^ (0.57 S/m) and 36% less RF absorption than physiological saline solution^29^ (1.54 S/m). We examined the RISHA signals collected from four polyethylene tubes (Fig. 2a) filled with vegetable oil, an aqueous solution of anticoagulant ethylenediamine tetraacetic acid (EDTA, 20.1341 µL/500 µL), mouse blood with the same EDTA concentration as the aqueous solution, or 0.9% physiological saline solution (see Methods for sample preparation). The RISHA image (Fig. 2b; rendered in red) shows the RF-absorbing contents of the tubes, which is not seen by ultrasonography (rendered in green). While the US image shows similar signals for all four tubes, RISHA responses vary as expected with electrical conductivity. We show in Fig. 2c the average RISHA/US responses along the *y*-axis of Fig. 2b, together with the standard deviation of the measurements. No RISHA signal was detected from the oil tube, since oil has negligible conductivity at 3.2 MHz, and relatively low RISHA signals were detected from the anticoagulant EDTA. Conversely, saline demonstrated the highest RISHA signals, and signals from blood showed an amplitude of ~85% of that of saline. The US images in these experiments (Fig. 2b) showed ‘shadows’, like the ones observed with copper wires (Fig. 1f), which we again attribute to narrowband US wave interference (see Supplementary Text 2 and Fig. S2).

To assess the sensitivity of the RISHA signals to the electrical conductivity differences between blood and surrounding tissue *ex vivo*, we imaged a synthetic experimental sample consisting of a polyethylene tube filled with mouse blood and overlaid with pieces of porcine fat and muscle tissue placed side-by-side (Fig. 2d). RISHA imaging (Fig. 2e,f) clearly resolves the blood and muscle tissue with the expected contrast of 6 dB (Fig. 2g), consistent with the conductivity-based differences of RF absorption. Because of its low conductivity^28^ (0.026 S/m), fat tissue was invisible on the RISHA image (Fig. 2e). The tubing and surrounding tissues were visible in the US image (Fig. 2f) because of their strong reflectivity of US waves. The yellow areas in Fig. 2f show the overlap of RISHA signals (red) and US reflections (green). The blood tube appeared thinner on the fat-tissue side in the RISHA image in Fig. 2e, whereas the US image in Fig. 2f indicates that the transducer remained focused along the tube. Therefore, the RISHA signal decrease is unlikely due to the angulated positioning of the tube relatively to the transducer aperture. Instead, this RISHA signal variation can be attributed to the natural sedimentation of red blood cells toward the bottom of the tube, where RF absorption decreases because red blood cells are less conductive than the blood plasma in the upper part of the tube^28,30^. While in Fig. 2b the transducer is focused on the rear wall of the tubing sample, in Fig. 2f the focus of transducer is placed to the center of the tube sample. Due to different foci alignments and the resulting interference of narrowband US waves, the shadow artifact is not visible in Fig. 2f (see also Supplementary Text 2 and Fig. S2).

We further explored whether RISHA imaging could resolve intrinsic contrast of vascular structures within chicken muscle *ex vivo*. A chicken tissue of ~3 mm thick containing a blood vessel (Fig. 2h) was imaged over a 14 mm × 5 mm field of view and clearly revealed an image of the blood vessel with 6-dB contrast between blood and muscle (Fig. 2i,k) and 500-µm resolution. Figure 2j shows the dual-mode RISHA/US image. The US image (green) shows practically no contrast between vasculature and surrounding muscle. Equivalent results were obtained from thicker (5 mm) and more heterogeneous *ex vivo* samples of chicken meat containing dermis, fat and muscle (see Supplementary Text 5 and Fig. S5). In these cases, RISHA imaging resolved vasculature located >3 mm below the surface with a TBR of 12 dB.

### RISHA imaging of mouse vasculature

**Figure 3:**
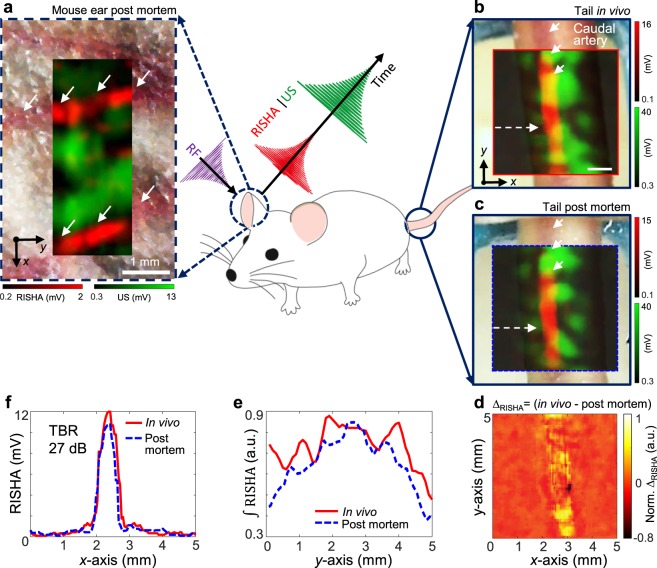
RISHA imaging of vasculature in living and euthanized mouse. An ear (dashed blue circle) of a mouse (illustrated in the center) was imaged after the mouse euthanasia, while the tail (solid blue circle) was imaged *in vivo* and then euthanized. (**a**) Co-registered RISHA (red) and US echo (green) images of an ear in a mouse post mortem, superimposed onto a microscopic photograph of the scanned area displaying small vessels. White arrows indicate blood vessels. (**b**) Co-registered RISHA/US images of mouse tail *in vivo*, superimposed onto the corresponding photograph. The red solid box shows the region scanned *in vivo*. Short white arrows indicate the caudal artery; the dashed arrow indicates the line profile analyzed in panel f for comparison of RF absorption. (**c**) Co-registered RISHA/US image of the same mouse tail as in panel b, but 30 mins after euthanasia of the animal. The dashed blue box shows the same scanned area as in panel *b*. Short white arrows indicate the caudal artery; the dashed arrow indicates the line profile shown in panel f. (**d**) The normalized difference map of RISHA response (arbitrary units, a.u.), ∆_RISHA_ = (*in vivo* - post mortem), obtained by subtracting the post-mortem signal from the pre-mortem signal, showing overall morphological changes. (**e**) The normalized integration $(\int {\rm{RISHA}})$ of RISHA signals in panels b and c along the *x*-axis. The resulting projection along the *y*-axis shows RF absorption by oxygenated blood in the mouse tail *in vivo* (solid red) and by deoxygenated blood after euthanasia (dashed blue). (**f**) Profiles of the lines scanned in RISHA images *in vivo* and post mortem in panels b and c, showing differences <10% in RISHA amplitude of 27 dB TBR. Scale bars are 1 mm.

To complement studies on synthetic phantoms and *ex vivo* tissue, we performed RISHA imaging of a mouse ear after euthanasia. Figure 3a shows the hybrid RISHA/US image overlaid on the microscopic photograph of the scanned mouse ear, revealing that RISHA imaging allows visualization of small vessels in mouse ear with a resolution of ~500 µm, consistent with both our experiments of resolution characterization using copper wire and the diffraction-limited resolution of RISHA imaging at 3.2-MHz RF excitation.

To study the change of blood in electrical conductivity in pre- and post-mortem animal, we also performed RISHA imaging in a mouse tail, both *in vivo* (Fig. 3b) and 30 minutes after mouse euthanasia when blood oxygenation level is low (Fig. 3c; see Methods for mouse experiment procedure). Overlay of co-registered RISHA/US images on the corresponding photographs of the mouse tail pre-mortem (Fig. 3b) and post-mortem (Fig. 3c) gave comparable results: RISHA signals (red) revealed the caudal artery beneath the skin, while US signals (green) provided complementary information about overall tail structure. In this arrangement, RISHA imaging did not detect the lateral or dorsal veins, because we only focused ultrasound detection on the middle caudal artery. An analysis of normalized RISHA signal differences between the pre- and post-mortem states (Fig. 3d–f) revealed amplitude differences of less than 10% between *in vivo* and post mortem, demonstrating that blood conductivity does not depend on blood oxygenation level or other post-mortem changes. Line profiles through the caudal artery in RISHA images *in vivo* and post mortem (Fig. 3f) indicate that the TBR in both RISHA images was above 27 dB, mainly due to the fact that the conductivity of blood in the caudal artery is higher than the conductivity of skin and bone.

## Discussion

We have presented a new label-free, non-invasive method to image conductivity paths within dielectric materials, including animal tissues. We showed that RF induces second harmonic acoustic responses and allows blood vessel imaging without the need to administer contrast agents. Imaging experiments *in vivo* and *ex vivo* showed that the RISHA response of blood, i.e. the blood conductivity, is independent of its oxygenation state. Our technique excites samples with RF in the high frequency band (3–30 MHz), and provides a final image resolution 2.2 × 10^4^ times smaller than the excitation wavelength of 10.5 meters. The use of near-field narrowband energy coupling maximizes RISHA response and minimizes energy loss. RISHA imaging requires only inexpensive, widely available RF hardware as well as widely available US detection hardware, enabling simultaneously US pulse-echo imaging of the sample without additional hardware. This dual-mode RISHA/US imaging method allows pixel-by-pixel co-registration of the conductivity path and sample morphology, which provides an intuitive, holistic visual understanding of the sample. Such a technique could be used not only for biomedical imaging, but also for imaging the interior of industrial products, such as integrated circuits, revealing both the conductive paths and morphology of the circuits.

RISHA vasculature imaging offers an alternative to other blood vessel imaging methods by using inexpensive and readily available components that can lead to a low-cost and highly portable sensor. Promising applications of portable RISHA imaging could be general detection of sub-dermal blood vessels for catheter insertion, or inexpensive, disseminated measurements of vasculature, for example in characterizing loss in response to anti-tumor therapies.

Our hybrid RISHA/US imaging system enabled dual contrast imaging which was optimized for blood vessel visualization. Better anatomical images using US imaging can be achieved by focusing the transducer on hyperechoic structures like tissue bone or using multiple ultrasound sensors with different focusing depth, or by using a dedicated US pulser/receiver unit as shown in Supplementary Fig. S1a,c.

RISHA imaging has a large dynamic range^15^ and can detect blood vessels with a TBR of more than 6 dB in muscle tissue and more than 27 dB in bone/skin tissues, providing a sensitive and non-invasive tool for assessing changes in blood conductivity^31^. Conductivity sensing could be more generally helpful in assessing bulk ionic content in body fluids, such as urine (1.75 S/m), where ion levels are an important marker of renal abnormalities^32,33^, offering diagnostic ability in bladder and kidney diseases. Other tissues could also be imaged using RISHA non-invasively aiding to disease diagnosis, including bile (1.4 S/m at 3 MHz), gallbladder (0.9 S/m), cerebrospinal fluid (2.00 S/m), and intervertebral disc (0.83 S/m), to name a few^28,34,35^.

It may be possible to bring the resolution of RISHA imaging into the micrometer range by using higher frequencies of acoustic detection (10–300 MHz), and the corresponding radiofrequency excitation (5–150 MHz) based on the second harmonic acoustic generation principle. While conductivity absorption remains as the dominant contrast^19^, RISHA of high resolution/frequency may allow label-free visualization of subcellular ion concentrations, analogous to what has been accomplished with optical/optoacoustic imaging^9^. This could make high-frequency RISHA an intriguing experimental tool for label-free cellular studies of ion transport and related processes. For example, RISHA can directly observe changes in ionic content of living cells in response to RF stimuli^36^. The ability to analyze electro-cellular^37,38^ and electro-thermo-cellular interactions may allow RISHA to enrich the bioengineering toolbox with a new, non-invasive electric field method analogous to optics^39^, patch-clamp^40^, or magnetics based techniques^41–43^.

Conventional pulsed thermoacoustic imaging at 434 MHz has been applied for breast cancer detection^44^, while higher excitation frequencies at several GHz have been used to image different biological tissues based on their dielectric losses^21–23,45^. These microwave-based thermoacoustic imaging methods provide good soft tissue contrast and the contrast could be further enhanced by administering contrast agents^22,46^. Nevertheless, shortcomings include low energy coupling due to far field radiation^17^, and poor imaging resolution >1 mm due to the long excitation pulses typically employed (pulse modulation >500 ns). RISHA addresses several limitations of existing thermoacoustic imaging methods by using near-field coupling and quasi-CW excitation in low-MHz range. However, our RISHA imaging system is limited by lack of contrast agents in the low-RF region^46,47^. The development of contrast agent for low frequency RF absorption is challenging and requires detailed understanding of the contrast mechanism in this frequency band^46,48^. Because RISHA imaging is based on inhomogeneous RF excitation in the reactive near-field of coupling elements^17,49^, future applications on thick samples larger than a mouse would require RF field strength correction^17,18^.

In summary, RISHA imaging demonstrated 6-dB blood-to-muscle contrast, high dynamic range of conductivity detection, and the ability to perform simultaneous RF-induced passive ultrasound imaging using inexpensive instrumentation. We demonstrated the ability to image at depths of up to 2.5 cm in muscle using RISHA imaging. Future system optimizations can improve the resolution and sensitivity, using optimized RF-field excitation and higher frequencies. RISHA imaging could be employed in various biomedical applications non-invasively assessing vasculature, overall tissue electrical conductivity, and also for functional imaging like characterization of the relationship between blood oxygenation and conductivity as well as thermoacoustic Doppler flowmetry of blood flow.

## Methods

### RISHA imaging set-up

The dual-mode RISHA/US imaging system (Fig. 1a) is driven by a custom-built quasi-continuous wave RF-field generator containing a RC discharging circuit and a custom-built helical coil for near-field energy coupling. The helical coil is wound with 3 mm diameter copper wire and shielded with heat shrink tubing (TF31-9/3, HellermannTyton) with dielectric strength of 37 kV/mm. The quality factor of the RF-field generator is 20, allowing the set-up to provide narrowband stimulation at 5% FWHM-bandwidth (0.16 MHz) and a central frequency of 3.2 MHz. A function generator (33210A, Agilent Technologies) controls the repetition rate of the quasi-continuous RF bursts (1–50 Hz, max 765 mJ per pulse). We performed raster scanning with a spherically focused transducer (V311, Olympus-NDT; central frequency, 10 MHz; focus, 25.4 mm) mounted on *xy*-translation stages (LTA-HS, ESP 300 controller, Newport Corp) that move across the sample in 100-µm steps in the *xy* plane corresponding to 1/5th of the diffraction limit of the 6.4 MHz RISHA wave. The coil, sample, and transducer are immersed in a de-ionized water bath to provide efficient US and RF coupling. To remove low frequency oscillation, we used a high-pass RC filter with cutoff frequency at 300 kHz to filter the signal detected by transducer. In our experiments, a 51-dB amplifier (AU-1332, MITEQ) amplified the signals in copper wire experiments, while a 63-dB amplifier (AU-1291, MITEQ) amplified the signals in experiments with biological tissues. The amplified signals were digitized by a digital oscilloscope (TDS3054B, Tektronix). The digitized data were averaged 16 or 32 times and stored on a personal computer, which was also used for synchronizing the raster scanning and for reconstructing the images.

In our *in vivo* measurement, we used 16 times averaged signals, with 50 Hz burst repetition rate. The raster scan step size was set to 100 μm. Considering 0.1 second for the data transmission and stage scan for each data point, the total imaging time for a 10 mm × 10 mm field of view is ~70 mins.

### Signal processing and image formation

Co-registered US and RISHA images can be reconstructed based on maximum intensity projections (MIPs) of the acquired acoustic signals: RISHA and US signals were distinguished based on their different frequency spectra and time of flight (Fig. 1b,c). In this work, in order to obtain optimal image target-to-background ratio, MIPs were generated based on the characteristic frequency-doubling in the Fourier spectra of the RISHA and US signals. The temporal sequences were first filtered by a low-pass filter with cutoff frequency of 20 MHz. The amplitude values of the Fourier spectrum at the frequency *f*_*RISHA*_ (6.4 MHz, for RISHA signal) or *f*_*US*_ (3.2 MHz, for US signal) were then used as the pixel value for each grid point to generate the RISHA and US images, correspondingly. Alternatively, if MIPs were generated in the time domain, a band-pass filter was first applied with a lower cutoff frequency of 2.7 MHz and higher cutoff of 3.7 MHz to the US data; and with 5.9 MHz and 6.9 MHz to the RISHA data, correspondingly. Then the images were filtered using a median filter, and the smallest pixel value was subtracted from all pixels to generate a dark background for better visualization. At each scanning point, RISHA and US signals were derived from the same acquired sequence, so co-registration of the two types of images was achieved simply by assigning the normalized RISHA image to the red channel of an RGB image, and the normalized US image to the green channel.

For better visualization of the mouse ear post mortem (Fig. 3a) and the chicken *ex vivo* results (Supplementary Fig. S5d,e), we applied a mild tubular-structure filter^50^ with Gaussian kernels of diameters ranging from 240 to 640 µm, corresponding to the imaging resolution range of our set-up.

### RISHA imaging resolution

The diffraction-limited lateral imaging resolution using RISHA (d_*RISHA*_) is defined by the RISHA wavelength (λ_*RISHA*_) and by the *f-*number of the ultrasound transducer, which is *F/D* (focus length *F* = 25.4 mm and diameter *D* = 12.7 mm). It can be calculated as:

3 $${{\rm{d}}}_{{RISHA}}={{\rm{\lambda }}}_{{RISHA}}\cdot (\frac{{F}}{{D}})=(\frac{{{v}}_{{s}}}{{{f}}_{{RISHA}}})\cdot (\frac{{F}}{{D}})=(\frac{{{v}}_{{s}}}{2\times {{f}}_{{RF}}})\cdot (\frac{{F}}{{D}})$$

where *v*_*s*_ is the speed of sound in water (1,480 m/s). With de-ionized water as the RF energy and acoustic coupling medium, the frequency of RF excitation relates to RF wavelength λ_*RF*_ as follows:

4 $${{f}}_{{RF}}=\frac{{{c}}_{0}/\sqrt{{{\varepsilon }}_{{r}}{\mu }_{{r}}}}{{{\rm{\lambda }}}_{{RF}}}$$

in which *c*_0_ is the speed of electromagnetic waves in a vacuum (3 × 10^8^ m/s), *ε*_*r*_ is relative permittivity of de-ionized water (80.1 at 20 °C), and *μ*_*r*_ is relative permeability of water (*μ*_*r*_ = 1). Based on Eq. (3) and Eq. (4), lateral resolution with RISHA imaging can be defined as:

5 $${{\rm{d}}}_{{RISHA}}=(\frac{{{v}}_{{s}}\cdot {{\rm{\lambda }}}_{{RF}}}{2\times {{c}}_{0}/\sqrt{{{\rm{\varepsilon }}}_{{r}}{\mu }_{{r}}}})\cdot (\frac{{F}}{{D}})$$

The achievable imaging resolution of RISHA appears to be a fixed fraction of the excitation RF wavelength employed. Substituting numbers into Eq. (5) yields a lateral resolution of 469 µm, corresponding to 1/(2.2 × 10^4^) of the RF wavelength at 3.2 MHz (according to Eq. (4), *λ*_*RF*_ = 10.5 meters). Axial resolution of RISHA imaging can then be calculated as 0.80 × λ_*RISHA*_ according to Wang *et al*.^51^, yielding a resolution of 188 µm.

### Sample preparation: Whole blood and chemical solutions

Polyethylene tubes (Fig. 2a) with a diameter of 3 mm were filled with vegetable oil, an aqueous solution of the anticoagulant ethylenediamine tetraacetic acid (EDTA, 20.1341 µL/500 µL), mouse blood^28^ (*σ* = 0.98 S/m) containing the same concentration of EDTA, or 0.9% physiological saline solution^29^ (NaCl, *σ* = 1.54 S/m). To ensure the same concentration of anticoagulant, we used the same type of EDTA anticoagulant microvette (Microvette 500 K3E, Sarstedt) to collect mouse blood and to prepare the tubes containing aqueous solution of EDTA.

### Sample preparation: Synthetic phantom and *ex vivo* tissue

A synthetic phantom was prepared by laying the polyethylene tube containing mouse blood on top of side-by-side pieces of porcine muscle and fat tissue *ex vivo* (Fig. 2d). Conductivity of muscle and fat are, 0.57 S/m and 0.026 S/m at 3 MHz and 20°C, respectively^28^. To preserve the ionic content of the porcine muscle and thereby its RF absorptivity, we covered it with low-density polyethylene film with a thickness of 20 µm, which shows similar acoustic impedance at 20 °C (*Z*_0_ = 1.73 µPa·s/mm) as water (*Z*_0_ = 1.48 µPa·s/mm). This kept acoustic losses below 0.7%. The low electrical conductivity of polyethylene^52^ (*σ* = 10^−15^ S/m) meant that RISHA responses at 3 MHz could be entirely attributed to the biological tissue (typically 0.01–1 S/m). The fat tissue in the phantom was not covered with polyethylene film because it was expected to show low RF absorption due to its low conductivity. The fat tissue can also be covered with same polyethylene film immersed in low RF-absorbing oil to preserve its ‘RF absorptivity’.

The *ex vivo* chicken soft tissue, shown in Fig. 2h, contained a blood vessel approximately 500 µm wide surrounded by muscle tissue. To conserve its RF absorptivity, this tissue was covered with the same polyethylene film as mentioned above.

### Sample preparation: Mouse vasculature experiments

All mouse procedures were approved by the Bavarian Animal Care and Use Committee and all experiments were performed in accordance with the guidelines and regulations approved by the Government of Bavaria, Germany. All imaging experiments have been performed with RF energy levels well within the safety standards defined by IEEE Standard for Safety Levels with Respect to Human Exposure to Radio Frequency Electromagnetic Fields, 3 kHz to 300 GHz^53^. The ear and tail of a CD-1 nude mouse were imaged using our dual-mode RISHA/US set-up. For *in vivo* measurements of the tail (Fig. 3b), the mouse was first anesthetized for 90 minutes by intraperitoneal injection of ketamine/xylazine (0.1 mL/g of mouse weight with 1 mL ketamine 100 mg/mL, 0.25 mL xylazine 20 mg/mL, and 6 mL physiology saline solution), then it was placed on a custom-built mouse bed above water level, and kept warm by an infrared lamp. During the experiment, the mouse tail was fixed to a 3D-printed imaging frame (blue PLA material in Fig. 3b,c) and immersed in de-ionized water. For tail imaging of the mouse after euthanasia (Fig. 3c), the animal was first injected intraperitoneally with 40 µL heparin (25 000 I.E./5 mL, Heparin-Natrium Braun) to keep blood from coagulating, and then the anesthetized mouse was sacrificed by cervical dislocation. While keeping the mouse in the same position on the mouse bed, we waited for 30 minutes to ensure low hemoglobin oxygenation level in blood. Then we scanned the tail again under the same conditions as for *in vivo* imaging. Upon finishing imaging of the mouse tail after euthanized, we changed the mouse position so that the ear was fixed to the imaging frame and scanned (Fig. 3a) under the same conditions as for the tail.

### k-Wave simulation

Acoustic interference that is caused by a tube with a diameter of 3 mm and wall thickness of 0.1 mm was simulated using the k-Wave Matlab toolbox^12^. The computational grid size was set to 0.1 mm, same as the mechanical scanning step size. The ultrasound transducer characteristics were as follows: central frequency, 10 MHz; bandwidth, 73.45%; focus distance, 25.4 mm; diameter, 12.7 mm. Mass density of polyethylene material was defined to be 940 kg/m^3^ and ultrasound speed to be 2080 m/s. The coupling medium was water, with a mass density of 998 kg/m^3^ and a speed of sound of 1,500 m/s. A time-varying, 3.2-MHz quasi-continuous US wave was specified for the transducer elements, and a time-varying RISHA wave of 6.4 MHz was specified for the aqueous content within the tube. The transducer was moved across the sample to simulate raster scanning. Signal processing and image reconstruction algorithms used in the simulation were similar to those used in imaging experiments. The analysis and result are shown in Supplementary Text 2 and Fig. S2.

## Electronic supplementary material

Supplementary Text 1-5 & Figure S1-5

## Data Availability

The raw data that support the findings of this study, and the Matlab programs that are used to collect, process and reconstruct the data are available from the corresponding authors on request.

## References

[1] Omar M, Schwarz M, Soliman D, Symvoulidis P, Ntziachristos V (2015). Pushing the optical imaging limits of cancer with multi-frequency-band raster-scan optoacoustic mesoscopy (RSOM). Neoplasia.

[2] Dronkers CE, Klok FA, Huisman MV (2016). Current and future perspectives in imaging of venous thromboembolism. J. Thromb. Haemost..

[3] Carmeliet P, Jain RK (2000). Angiogenesis in cancer and other diseases. Nature.

[4] Pandian NG (1988). Ultrasound angioscopy: real-time, two-dimensional, intraluminal ultrasound imaging of blood vessels. Am. J. Cardiol..

[5] Errico C (2015). Ultrafast ultrasound localization microscopy for deep super-resolution vascular imaging. Nature.

[6] Camici PG, d’Amati G, Rimoldi O (2015). Coronary microvascular dysfunction: mechanisms and functional assessment. Nat. Rev. Cardiol..

[7] Taruttis A (2016). Optoacoustic Imaging of Human Vasculature: Feasibility by Using a Handheld Probe. Radiology.

[8] Taruttis A, Ntziachristos V (2015). Advances in real-time multispectral optoacoustic imaging and its applications. Nat. Photonics.

[9] Omar M, Soliman D, Gateau J, Ntziachristos V (2014). Ultrawideband reflection-mode optoacoustic mesoscopy. Opt. Lett..

[10] Aguirre J (2017). Precision assessment of label-free psoriasis biomarkers with ultra-broadband optoacoustic mesoscopy. Nat. Biomed. Eng..

[11] Huang D (1991). Optical coherence tomography. Science.

[12] Treeby BE, Cox BT (2010). k-Wave: MATLAB toolbox for the simulation and reconstruction of photoacoustic wave fields. J. Biomed. Opt..

[13] Chugh BP (2009). Measurement of cerebral blood volume in mouse brain regions using micro-computed tomography. Neuroimage.

[14] Lentschig MG (1998). Breath-hold gadolinium-enhanced MR angiography of the major vessels at 1.0 T: dose-response findings and angiographic correlation. Radiology.

[15] Kellnberger S, Omar M, Sergiadis G, Ntziachristos V (2013). Second harmonic acoustic responses induced in matter by quasi continuous radiofrequency fields. Appl. Phys. Lett..

[16] Kellnberger S (2016). Magnetoacoustic Sensing of Magnetic Nanoparticles. Phys. Rev. Lett..

[17] Razansky D, Kellnberger S, Ntziachristos V (2010). Near-field radiofrequency thermoacoustic tomography with impulse excitation. Med. phys..

[18] Kellnberger S, Hajiaboli A, Razansky D, Ntziachristos V (2011). Near-field thermoacoustic tomography of small animals. Phys. Med. Biol..

[19] Omar M, Kellnberger S, Sergiadis G, Razansky D, Ntziachristos V (2012). Near-field thermoacoustic imaging with transmission line pulsers. Med. phys..

[20] Bowen, T. In *1981 Ultrasonics Symposium* 817–822 (IEEE, Chicago, IL, USA, 1981).

[21] Ku G, Wang LV (2001). Scanning microwave-induced thermoacoustic tomography: signal, resolution, and contrast. Med. phys..

[22] Alireza M, John HB, Susan CH (2009). Toward contrast-enhanced microwave-induced thermoacoustic imaging of breast cancer: An experimental study of the effects of microbubbles on simple thermoacoustic targets. Phys. Med. Biol..

[23] Aliroteh MS, Arbabian A (2018). Microwave-Induced Thermoacoustic Imaging of Subcutaneous Vasculature With Near-Field RF Excitation. IEEE Trans. Microw. Theory Tech..

[24] Ding WZ, Ji Z, Da XING (2017). Microwave-excited ultrasound and thermoacoustic dual imaging. Appl. Phys. Lett..

[25] Mohajerani P, Kellnberger S, Ntziachristos V (2014). Frequency domain optoacoustic tomography using amplitude and phase. Photoacoustics.

[26] Soliman D, Tserevelakis GJ, Omar M, Ntziachristos V (2015). Combining microscopy with mesoscopy using optical and optoacoustic label-free modes. Sci. Rep..

[27] Beard P (2011). Biomedical photoacoustic imaging. Interface Focus.

[28] Gabriel S, Lau RW, Gabriel C (1996). The dielectric properties of biological tissues: II. Measurements in the frequency range 10 Hz to 20 GHz. Phys. Med. Biol..

[29] Mazzoleni AP, Sisken BF, Kahler RL (1986). Conductivity values of tissue culture medium from 20 °C to 40 °C. Bioelectromagnetics.

[30] Hirsch FG (1950). The electrical conductivity of blood. I: Relationship to erythrocyte concentration. Blood.

[31] Abdalla S, Al-Ameer SS, Al-Magaishi SH (2010). Electrical properties with relaxation through human blood. Biomicrofluidics.

[32] Wang JM (2014). Evaluating the performance of urine conductivity as screening for early stage chronic kidney disease. Clin. Lab..

[33] Manoni F (2009). Laboratory diagnosis of renal failure: urine conductivity and tubular function. Minerva Urol. Nefrol..

[34] Gabriel S, Lau RW, Gabriel C (1996). The dielectric properties of biological tissues: III. Parametric models for the dielectric spectrum of tissues. Phys. Med. Biol..

[35] Hasgall, P. *et al*. *IT’IS Database for thermal and electromagnetic parameters of biological tissues*, https://www.itis.ethz.ch/database (2018).

[36] Grossman N (2017). Noninvasive Deep Brain Stimulation via Temporally Interfering Electric Fields. Cell.

[37] Bassett CA, Pawluk RJ (1972). Electrical behavior of cartilage during loading. Science.

[38] Shamos MH, Lavine LS (1967). Piezoelectricity as a Fundamental Property of Biological Tissues. Nature.

[39] Boyden ES, Zhang F, Bamberg E, Nagel G, Deisseroth K (2005). Millisecond-timescale, genetically targeted optical control of neural activity. Nat. Neurosci..

[40] Mainen ZF, Sejnowski TJ (1995). Reliability of spike timing in neocortical neurons. Science.

[41] Munshi R (2017). Magnetothermal genetic deep brain stimulation of motor behaviors in awake, freely moving mice. Elife.

[42] Drude P (1900). Zur elektronentheorie der metalle; II. Teil. galvanomagnetische und thermomagnetische effecte. Ann. Phys. (Berl.).

[43] Drude P (1900). Zur elektronentheorie der metalle; I. Teil. Ann. Phys. (Berl.).

[44] Kruger RA (2000). Breast cancer *in vivo*: contrast enhancement with thermoacoustic CT at 434 MHz-feasibility study. Radiology.

[45] Nie L, Xing D, Zhou Q, Yang D, Guo H (2008). Microwave-induced thermoacoustic scanning CT for high-contrast and noninvasive breast cancer imaging. Med. phys..

[46] Ogunlade O, Beard P (2015). Exogenous contrast agents for thermoacoustic imaging: an investigation into the underlying sources of contrast. Med. phys..

[47] Tamarov K (2017). Electrolytic conductivity-related radiofrequency heating of aqueous suspensions of nanoparticles for biomedicine. Phys. Chem. Chem. Phys..

[48] Pramanik M, Swierczewska M, Green D, Sitharaman B, Wang LV (2009). Single-walled carbon nanotubes as a multimodal-thermoacoustic and photoacoustic-contrast agent. J. Biomed. Opt..

[49] Nikitin, P. V., Rao, K. V. S. & Lazar, S. In 2007 IEEE *International Conference on RFID*. 167–174 (2007).

[50] Jerman T, Pernus F, Likar B, Spiclin Z (2016). Enhancement of Vascular Structures in 3D and 2D Angiographic Images. IEEE Trans. Med. Imaging.

[51] Xu MH, Wang LHV (2006). Photoacoustic imaging in biomedicine. Rev. Sci. Instrum..

[52] Adamec V, Calderwood JH (1981). On the determination of electrical conductivity in polyethylene. J. Phys. D: Appl. Phys..

[53] IEEE Standard for Safety Levels with Respect to Human Exposure to Radio Frequency Electromagnetic Fields, 3 kHz to 300 GHz, 10.1109/IEEESTD.2006.99501 (2006).

