# Supplementary material for: Blood vessel imaging using radiofrequency-induced second harmonic acoustic response
Source: Sci Rep. 2018 Oct 19;8:15522. doi: 10.1038/s41598-018-33732-0 (PMC6195590; doi:10.1038/s41598-018-33732-0)
Supplement: Supplementary file 1 — Supplementary Text 1-5 & Figure S1-5 [file 41598_2018_33732_MOESM1_ESM.docx]

Supplementary Information for

# Blood vessel imaging using radiofrequency-induced second harmonic acoustic response

Yuanhui Huang^+^, Stephan Kellnberger^+,*^, George Sergiadis, Vasilis Ntziachristos^*^

^+^Y.H.H. and S.K. contributed equally to this work.

^*^Correspondence should be addressed to S.K. (stephan.kellnberger@tum.de) or to V.N. (v.ntziachristos@tum.de).

This SI file includes:

**Supplementary Text 1. Ultrasound signal amplitude comparison between conventional pulse/echo ultrasound and US signals generated by our RF-induced source.**

**Supplementary Text 2. Simulation of narrowband acoustic interference.**

**Supplementary Text 3. Depth resolving and axial resolution of RISHA imaging.**

**Supplementary Text 4. RISHA imaging depth in muscle tissue.**

**Supplementary Text 5. RISHA imaging of heterogeneous tissue *ex vivo*.**

**Supplementary Figure S1. Ultrasound signal amplitude comparison between conventional pulse/echo ultrasound and US signals generated by our RF-induced source.**

**Supplementary Figure S2. K-Wave simulation study of narrowband acoustic interference.**

**Supplementary Figure S3. Depth resolving and axial resolution of RISHA imaging.**

**Supplementary Figure S4. RISHA imaging in deep muscle tissue.**

**Supplementary Figure S5. RISHA imaging of heterogeneous biological tissue *ex vivo*.**

Supplementary Text

Supplementary Text 1. Ultrasound signal amplitude comparison between conventional pulse/echo ultrasound and US signals generated by our RF-induced source.

We performed experiments to quantitatively compare the US signal strengths generated by RISHA to an US wave generated with a commercially available pulser/receiver unit (Pulser/Receiver unit 5077PR, Olympus-Panametrics, USA).

In these experiments, we used a focused ultrasound transducer (V311, central frequency, 10 MHz; bandwidth, 73.45%; focus distance, 25.4 mm; diameter, 12.7 mm, Olympus-NDT, USA) which we also employed in our RISHA imaging studies. For conventional US generation, the transducer is driven by the pulser/receiver unit with a voltage amplitude of 200 Volt. In the RF-induced US generation experiment, the transducer is driven by energy emitted from the 3.2 MHz RF pulser used for RISHA signal generation.

In a first set of experiments shown in Supplementary Fig. S1a, we quantitatively compared the transducer relaxation signal amplitude from the pulser/receiver unit (Experiment 1, dashed blue line) with the US-signal generated by the RISHA setup (Experiment 2, solid green line).
Experiment 1 - Pulser/receiver signal amplitude: Using a 200 V pulse amplitude, we measured a transducer relaxation amplitude of 743.3 mV based on -49 dB signal attenuation. This signal corresponds to the pulse amplitude of 743.3×10^-3^ Volt × 281.8 = 209.5 Volt generated by the pulser/receiver.
Experiment 2 - RISHA excitation signal amplitude: Using RISHA excitation pulses with 765 millijoule (mJ) per pulse, we measured a transducer relaxation (RF-induced US emission) peak amplitude of -99.6 mV.

According to this experiment, the difference between conventional pulser/receiver generated US and RF-induced US is 66 dB, or a factor of 2.1×10^3^.

We further investigated US signal amplitudes of the pulser/receiver and the RISHA setup in pulse/echo mode on an ultrasound phantom consisting of two tubes. Supplementary Fig. S1b shows the phantom used for this study and Supplementary Fig. S1c,d displays the experimental results of the B-scan. When using the conventional US pulser/receiver unit, we recorded a peak US-echo amplitude of 73.9 mV, without any amplification or attenuation. When using RF-induced US generation, we recorded a peak US-echo amplitude of 65.2 mV, but with a 63-dB amplification (increased by 1412.5 times in voltage, AU-1291, MITEQ), corresponding to a maximum amplitude of RF-induced US echo of 65.2 mV ÷ 1412.5 = 46.2 µV.

This experimental comparison yields a difference of 1.6×10^3^, corresponding to 64 dB, between conventional US and RF-induced US, being in the same order as the transducer relaxation experiment.

**Supplementary Text 2. Simulation of narrowband acoustic interference.**

Supplementary Fig. S2a,b shows the misalignment appearing as ‘shadow’ from experimental dual-mode RISHA/US image of blood tubing in Fig. 2b, which we attribute to the narrowband characteristics of the transmitted US wave and possibly to destructive and constructive interferences of US waves back-reflected from the sample. To test this hypothesis, we used the k-Wave toolbox (see Methods) to demonstrate that when the transducer is focused on the rear surface of the tubing, the simulated dual-mode image (Supplementary Fig. S2c) is similar to the experimental blood tubing image (Supplementary Fig. S2a). The simulated line profile (Supplementary Fig. S2d) is like the experimental one (Supplementary Fig. S2b), indicating a shift of approximately 300 µm between the RISHA and US signal peaks. Therefore, we suggest that the acoustic interference of narrowband excitation at 3.2 and 6.4 MHz causes the visual ‘shadow’ effect in the dual-mode RISHA/US images.

While in Fig. 2b the transducer is focused on the rear wall of the tubing sample, in Fig. 2f the focus of transducer is placed to the center of the tube sample. Due to different foci alignments and the resulting interference of narrowband US waves, the shadow artifact is not visible in Fig. 2f.

The simulation shows also side lobes in the RISHA signal (Supplementary Fig. S2c,d), which coincide with those that we observed in our copper wire experiments for determining imaging resolution (Fig. 1e,h). Therefore, we suggest that these side lobes also result from constructive and destructive interference of RISHA waves at 6.4 MHz.

Acoustic interference, in addition to explaining the ‘shadow’ effect and the appearance of side lobes, also explains why the full width at half maximum of our copper wire phantom in US images (~1.5 mm) appears 50% larger than the diffraction-limited resolution of 940 µm predicted for the transducer at 3.2 MHz (Fig. 1f). Therefore, acoustic interference affects the apparent US lateral resolution in US imaging mode. It may be possible to reduce these interference effects by including more frequency components when exciting samples and when reconstructing RISHA and US images.

Supplementary Text 3. Depth resolving and axial resolution of RISHA imaging.

As shown in Fig. 1b,c, RISHA enables spatial differentiation of objects in the axial direction based on time-of-flight measurements, similar to conventional time-domain optoacoustic imaging.

Firstly, to show the spatial differentiation of objects, we performed RISHA imaging on the tube samples shown in Supplementary Fig. S1b. The results are shown in Supplementary Fig. S3a,b, demonstrating that RISHA can clearly resolve the four walls of the two tube samples in axial direction.

In a second experiment, we quantitatively characterized the axial resolution of our RISHA imaging setup using the thin copper wire samples (diameter: ∅200 µm) used in Fig. 1d-h. The reconstructed RISHA B-scan of the wire is shown in Supplementary Fig. S3c, while Supplementary Fig. S3d shows the line profile of the wire indicated by the red arrow in Supplementary Fig. S3c. From the peak envelope function of the RISHA burst, we calculated a full-width at half-maximum (FWHM) of 0.9 µs which corresponds to approximately 1.35 mm.

Correspondingly, applying deconvolution of the imaged object^1^, we calculated the axial resolution of the current RISHA system to 1.348 mm.

We note that, due to the relatively long duration of the RF excitation burst^2^, the axial resolution of RISHA is currently limited to 1.35 mm. However, future improvements such as shorter bursts and higher excitation frequencies^3-5^ can improve resolution.

Supplementary Text 4. RISHA imaging depth in muscle tissue.

To investigate the depth-penetration of RISHA imaging using low-MHz near-field RF energy, we constructed phantoms consisting of a copper wire inserted at different depths within *ex vivo* chicken muscle tissue (Supplementary Fig. S4a,b). A copper wire of 1-mm diameter was used as a reference for non-soft tissue penetration (0 mm penetration). Two phantoms were constructed (Supplementary Fig. S4a): phantom 1 consisted of the same copper wire inserted into the center of a piece of chicken muscle approximately 20 mm thick, resulting in propagation distances of 10 mm for incoming RF energy, 10 mm for outgoing RISHA waves, and 20 mm for roundtrip propagation of US waves in tissue; phantom 2 was constructed with the same copper wire as phantom 1, but with muscle tissue approximately 45 mm thick, giving a propagation distance of 25 mm for incoming RF energy, 20 mm for outgoing RISHA waves, and 40 mm for US wave roundtrip in tissue.

We performed RISHA/US imaging of these phantoms and observed loss of image quality with deeper penetration. Supplementary Fig. S4c shows dual-mode RISHA/US imaging of the reference copper wire in the absence of tissue, providing optimal imaging at “zero” penetration depth in muscle. Supplementary Fig. S4d shows copper imaging with the 20-mm thick tissue in phantom 1; Supplementary Fig. S4e shows the results with the 45-mm thick tissue in phantom 2. The loss of image quality with increasing penetration depth was confirmed by RISHA line profiles along the *x-*axis, as shown in Supplementary Fig. S4f. However, RISHA imaging maintained a target-to-background ratio (TBR) of approximately 6 dB for copper in the 45-mm muscle tissue. Supplementary Fig. S4g shows that the TBR of the corresponding US images depends less on depth. Although the frequencies of RISHA waves (6.4 MHz) and US waves (3.2 MHz) detected in these images have a ratio of 2/1, the discrepancy in TBR between RISHA and US imaging is not likely to be a result of frequency-dependent ultrasound attenuation, which is empirically known to be 0.75 dB/cm/MHz in soft tissue^6^, when the ratio of distances traveled by RISHA and US waves is considered.

These experimental results suggest that RF energy losses and the near-field distribution characteristics of the RF energy are the key determinants of penetration depth during RISHA imaging of thick conductive tissue. In its current state, the RISHA set-up allows imaging of conductive material at a TBR of 6 dB to a depth of 25 mm in muscle tissue.

Supplementary Text 5. RISHA imaging of heterogeneous tissue *ex vivo*.

In addition to imaging homogeneous chicken muscle, we also imaged more complicated, heterogeneous tissues (see Methods for sample preparation). These experiments demonstrate the rich contrast for differentiating conductive/RF-absorbing tissues at depths greater than several millimeters using RISHA imaging.

We firstly imaged chicken dermis ~5 mm thick (Supplementary Fig. S5a-c). To prevent ionic content of tissue leaching into de-ionized water, we immersed the samples in low RF-absorbing oil and wrapped them in polyethylene film. Supplementary Fig. S5c shows the dual-mode RISHA/US image with the transducer focused beneath the skin, revealing rich details of tissue heterogeneity, potentially corresponding to microstructures such as blood vessels or capillaries, glands, and other structures. The RISHA image (in red) shows tubular structures that are likely to be blood capillaries, while the corresponding US image (in green) shows skin folds of different ultrasound reflectivity. In another chicken muscle sample of >5 mm thickness (Supplementary Fig. S5d,e), RISHA imaging reveals similar tubular blood vessels at a depth (>3 mm) where the vessels are invisible in the optical photograph (Supplementary Fig. S5e).

Supplementary Figures


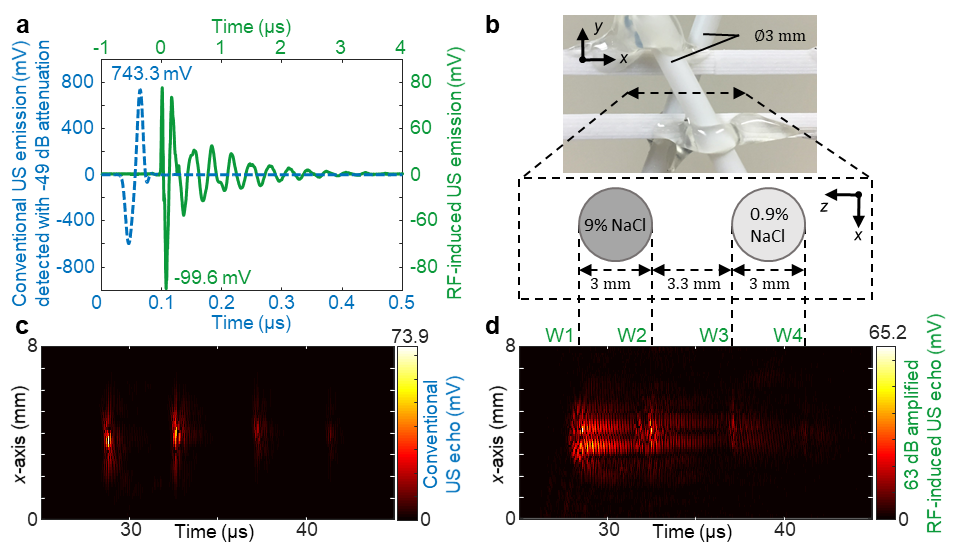


**Supplementary Figure S1. Ultrasound signal amplitude comparison between conventional pulse/echo ultrasound and US signals generated by our RF-induced source.** (**a**) Ultrasound emission amplitude comparison between a commercial pulser/receiver unit (dashed blue, conventional US emission, -49 dB attenuated) and the RF-induced method (solid green, RF-induced US emission). (**b-d**) Experimental results showing comparison of the ultrasound echo amplitude generated by (c) conventional and (d) our RF-induced source. (**b**) Photograph of two tubing samples (outer diameter ∅3 mm, wall thickness 100 µm) that were used in US pulse/echo imaging experiment. The tubes were filled with 9% and 0.9 % NaCl saline solution. The samples were fixated onto a 3D-printed holder and separated in axial direction (*z*-axis) by 3.3 mm. The focus of ultrasound transducer (focal length 25.4 mm, US echo appears at 33.8 µs) was positioned between the two tubing samples. (**c**) US B-scan result using conventional pulse/echo ultrasound. The maximum US echo amplitude recorded without amplification is 73.9 mV. (**d**) US B-scan using RF-induced US generation by RISHA setup. The maximum RF-induced US echo amplitude recorded with 63 dB amplification is 65.2 mV. W1-4 indicate the temporal position of the four walls of the tubes in US echo sequence.


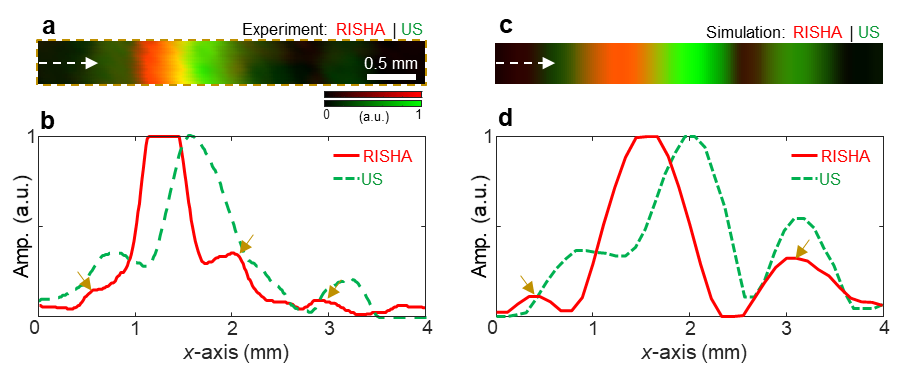


**Supplementary Figure S2.** **k-Wave simulation study of narrowband acoustic interference.** (**a**) Enlarged experimental RISHA/US image of blood tubing area enclosed in the dashed brown box in Fig. 2b, showing the ‘shadow’ effect on the US image (rendered in green). The red channel is the RISHA image. The dashed arrow indicates the line profiled in panel b. (**b**) Profiles of RISHA signal and US signal in experimental image of tubing sample. (**c-d**) k-Wave simulation of RISHA/US imaging of tubing with a diameter of 3 mm containing conductive sample, like the experimental phantom of tubing containing blood. (**c**) Simulated dual-mode RISHA/US image showing similar ‘shadow’ effect acquired when the focus of the transducer was set to the rear surface of the tubing sample. The dashed arrow indicates the line profiled in panel d. (**d**) Simulated profiles of RISHA signal and US signal along the arrow in panel c, showing lateral displacement of ~300 µm due to acoustic interference. Brown arrows indicate side lobes, which are also visible in the experimental RISHA images of copper wires in Fig. 1e,h. Scale bar, 0.5 mm.


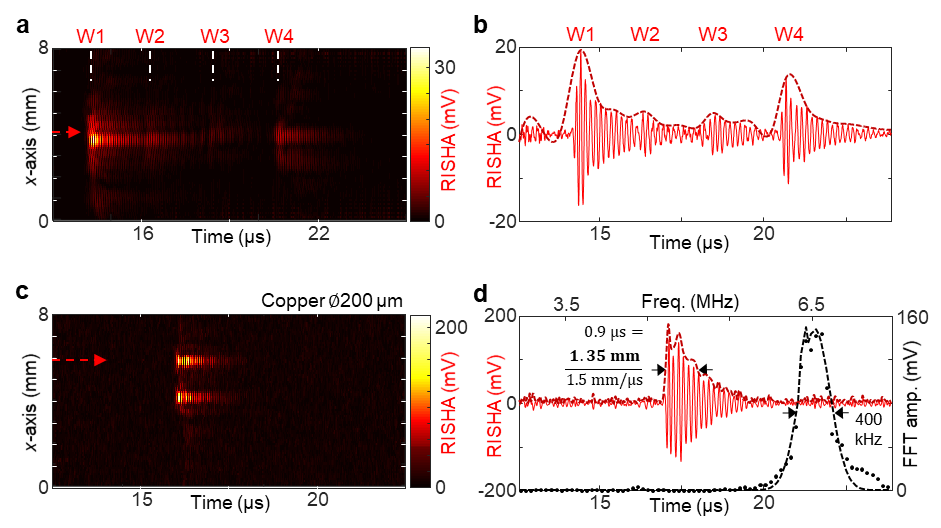


**Supplementary Figure S3. Depth resolving and axial resolution of RISHA imaging.** (**a-b**) Depth-resolution performance of RISHA imaging. (**a**) RISHA B-scan of tube samples as shown in Supplementary Fig. S1, showing the ability of RISHA to resolve the tubes in the axial direction. The focus of ultrasound transducer (RISHA signal appears at 16.9 µs) was positioned between the two tubing samples. The dashed white line segments indicate in axial direction the position of the four walls (W1-4) of the tubing samples. The dashed red arrow indicates the line profile shown in panel b. (**b**) The temporal waveform of RISHA signal acquired at position indicated by arrow in panel a. The temporal waveform shows the time-of-flight difference of four walls of the two tube samples as indicated by location of W1-4 corresponding to the signal peak envelope. (**c-d**) RISHA axial resolution characterization. (**c**) RISHA B-scan of the copper wires sample with diameter ∅200 µm along the dashed white arrow in Fig. 1e. The dashed red arrow indicates the line profile analyzed in panel d. (**d**) The temporal waveform of RISHA signal (solid red) acquired at position indicated by arrow in panel c. The RISHA burst duration is estimated from the peak envelope of the RISHA signal (dashed dark red) to be 3.3 µs; the FWHM of the RISHA burst duration is then estimated from the peak envelope function to be 0.9 µs, corresponding to a size of 1.35 mm given speed of sound of 1.5 mm/µs. The Fourier transform (FFT, dashed black curve fitted using FFT data points) shows the central frequency (6.5 MHz) and FWHM bandwidth (400 kHz) of the RISHA signal.


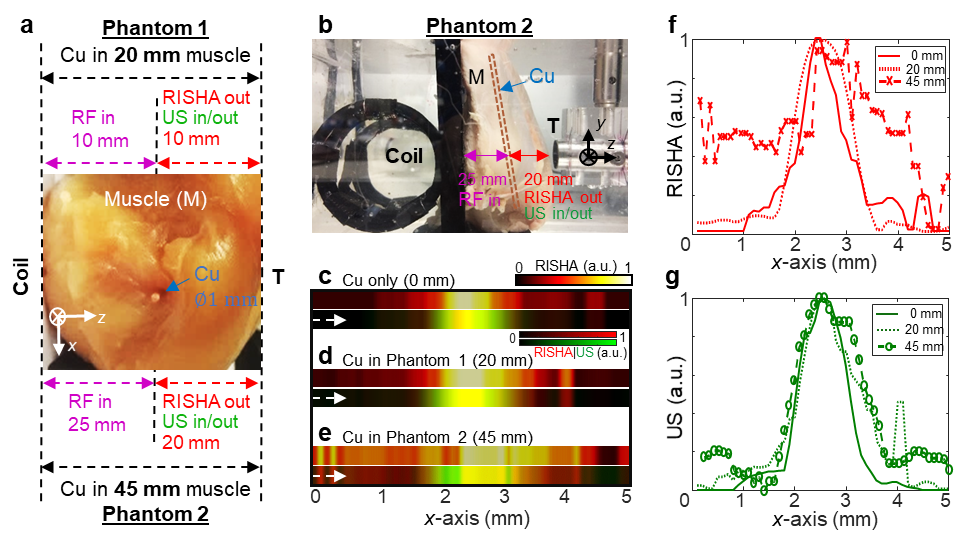


**Supplementary Figure S4. RISHA imaging in deep muscle tissue.** (**a**) Cross-sectional photograph of the phantoms showing penetration depth. As indicated, phantoms were placed between the energy coupling coil and the ultrasound transducer (T). Phantom 1 comprised chicken muscle (M) ~20 mm thick containing in its center a copper wire (Cu, ∅1 mm, blue arrow), providing 10 mm RF penetration depth, 10 mm RISHA wave travel distance to transducer, and 20 mm roundtrip US travel distance in tissue. Phantom 2 comprised chicken muscle ~45 mm thick containing in its center the same copper wire, providing 25 mm RF penetration depth, 20 mm RISHA wave travel distance, and 40 mm roundtrip US travel distance in tissue. The photograph shows phantom 2. (**b**) Photograph of phantom 2 when installed in imaging chamber, with the copper wire (Cu) artificially illustrated as a dashed brown rectangle. (**c-e**) RISHA images, and co-registered RISHA/US images with RISHA (red) and US (green) responses of copper wire imaged (**c**) in the absence of any muscle (0 mm), (**d**) in 20 mm thick muscle tissue (phantom 1), and (**e**) in 45 mm thick muscle tissue (phantom 2). Dashed arrows indicate line profiles analyzed in panels f and g. (**f**) RISHA signal profiles of copper imaged without any muscle (0 mm) or in 20 or 45 mm muscle tissue, along the arrows in panels c-e, respectively. (**g**) US echo signal profiles of copper imaged without any muscle (0 mm) or in 20 or 45 mm muscle tissue, along the arrows in panels c-e, respectively.


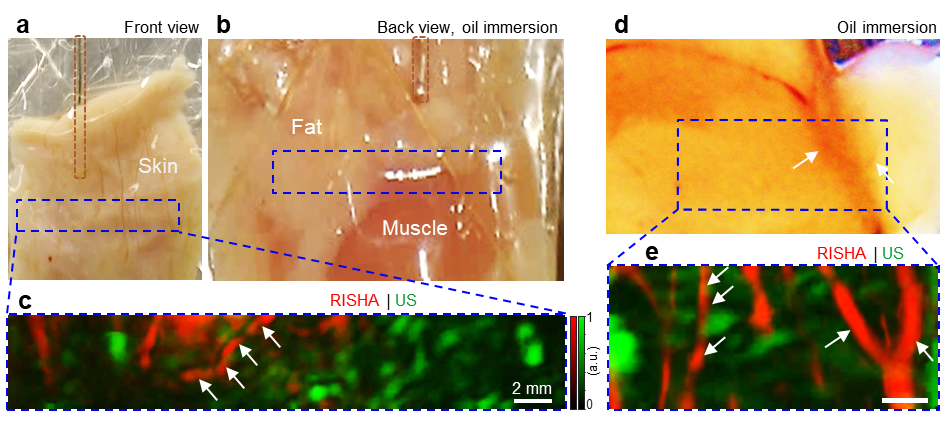


**Supplementary Figure S5.** **RISHA imaging of heterogeneous biological tissue *ex vivo*.** (**a-c**) RISHA/US imaging on a sample consisting of chicken dermis tissue approximately 5 mm thick containing skin, fat, muscle, blood capillaries, as well as a copper wire as positioning reference (indicated by dashed brown box). The dermis sample is shown in panel (**a**) front view/transducer view, and (**b**) back view. The sample was immersed in vegetable oil and wrapped in polyethylene film to preserve conductivity/RF absorptivity. The dashed blue box indicates the area scanned. (**c**) Co-registered RISHA/US image of the sample area as indicated in panel a, with RISHA signals rendered in red and US signals rendered in green. White arrows indicate vasculature. (**d-e**) RISHA/US image of a sample of chicken muscle >5 mm thick containing blood vessels (white arrows). The sample was immersed in vegetable oil and wrapped in polyethylene film to preserve conductivity/RF absorptivity. The blue dashed box indicates the area scanned. (**d**) Photograph of the thick muscle sample. (**e**) Co-registered RISHA/US image of the area within the dashed blue box in panel d. Tubular structures likely corresponding to blood vessels (arrows). Scale bars, 2 mm.

References

1 Soliman, D., Tserevelakis, G. J., Omar, M. & Ntziachristos, V. Combining microscopy with mesoscopy using optical and optoacoustic label-free modes. *Sci. Rep.* **5**, 12902; 10.1038/srep12902 (2015).

2 Kellnberger, S., Omar, M., Sergiadis, G. & Ntziachristos, V. Second harmonic acoustic responses induced in matter by quasi continuous radiofrequency fields. *Appl. Phys. Lett.* **103**, 153706 (2013).

3 Razansky, D., Kellnberger, S. & Ntziachristos, V. Near-field radiofrequency thermoacoustic tomography with impulse excitation. *Med. Phys.* **37**, 4602-4607 (2010).

4 Kellnberger, S., Hajiaboli, A., Razansky, D. & Ntziachristos, V. Near-field thermoacoustic tomography of small animals. *Phys. Med. Biol.* **56**, 3433-3444 (2011).

5 Omar, M., Kellnberger, S., Sergiadis, G., Razansky, D. & Ntziachristos, V. Near-field thermoacoustic imaging with transmission line pulsers. *Med. Phys.* **39**, 4460-4466 (2012).

6 Beard, P. Biomedical photoacoustic imaging. *Interface Focus* **1**, 602-631 (2011).
